# The Role of CD4^+^ Resident Memory T Cells in Local Immunity in the Mucosal Tissue – Protection Versus Pathology –

**DOI:** 10.3389/fimmu.2021.616309

**Published:** 2021-04-21

**Authors:** Kiyoshi Hirahara, Kota Kokubo, Ami Aoki, Masahiro Kiuchi, Toshinori Nakayama

**Affiliations:** ^1^ Department of Immunology, Graduate School of Medicine, Chiba University, Chiba, Japan; ^2^ AMED-PRIME, Japan Agency for Medical Research and Development, Chiba, Japan; ^3^ AMED-CREST, Japan Agency for Medical Research and Development, Chiba, Japan

**Keywords:** CD4+ resident memory T cells, Aspergillus fumigatus, lung fibrosis, ATAC-seq, inducible bronchus-associated lymphoid tissue (iBALT), pathogenic T cell

## Abstract

Memory T cells are crucial for both local and systemic protection against pathogens over a long period of time. Three major subsets of memory T cells; effector memory T (T_EM_) cells, central memory T (T_CM_) cells, and tissue-resident memory T (T_RM_) cells have been identified. The most recently identified subset, T_RM_ cells, is characterized by the expression of the C-type lectin CD69 and/or the integrin CD103. T_RM_ cells persist locally at sites of mucosal tissue, such as the lung, where they provide frontline defense against various pathogens. Importantly, however, T_RM_ cells are also involved in shaping the pathology of inflammatory diseases. A number of pioneering studies revealed important roles of CD8^+^ T_RM_ cells, particularly those in the local control of viral infection. However, the protective function and pathogenic role of CD4^+^ T_RM_ cells that reside within the mucosal tissue remain largely unknown. In this review, we discuss the ambivalent feature of CD4^+^ T_RM_ cells in the protective and pathological immune responses. We also review the transcriptional and epigenetic characteristics of CD4^+^ T_RM_ cells in the lung that have been elucidated by recent technical approaches. A better understanding of the function of CD4^+^ T_RM_ cells is crucial for the development of both effective vaccination against pathogens and new therapeutic strategies for intractable inflammatory diseases, such as inflammatory bowel diseases and chronic allergic diseases.

## What Are Tissue-Resident Memory T Cells?

“Immune memory” is a central and characteristic phenomenon of the acquired immune system. The long-term survival of the antigen-specific memory T cell population in response to invading harmful microorganisms is essential for the establishment of immune memory *in vivo*. Memory T cells can respond directly and rapidly to re-invading harmful microorganisms and efficiently eliminate them to protect the host.

Memory T cells were originally classified into two subpopulations, effector memory T (T_EM_) cells and central memory T (T_CM_) cells, based on (1) the expression pattern of cell surface molecules, (2) the orientation to specific tissues and (3) responsiveness to re-stimulation with a certain antigen (1). T_EM_ cells show the low expression of CCR7, a chemokine receptor that is crucial for homing to the secondary lymphoid organ and the low expression of the cell surface molecule CD62L. T_EM_ cells are mainly found in the non-lymphoid tissues and are responsible for peripheral immune surveillance and the immediate protective function in the host. T_EM_ cells respond quickly to re-stimulation of antigens and produce large amounts of proinflammatory cytokines, including IFN-γ, IL-5 and IL-4, but they showed shortened telomeres (2). In contrast, T_CM_ cells highly express both CCR7 and CD62L and migrate to sites with secondary lymphoid tissues, such as lymph nodes; T_CM_ cells primarily produce IL-2 upon antigen restimulation. After proliferation, T_CM_ cells efficiently produce large amounts of proinflammatory cytokines, such as IFN-γ and IL-4 (3, 4). Memory T cells are subdivided by various cell-surface markers, including CD27, CD127, CD43, CXCR3 and CX3CR1 (5–8). A study using CX3CR1-reporter mice reveals that CX3CR1^hi^ CD8^+^ T_EM_ cells were largely excluded from peripheral tissues after viral infection, providing novel insight concerning CD8^+^ T_EM_ cells (9).

**Figure 1:**
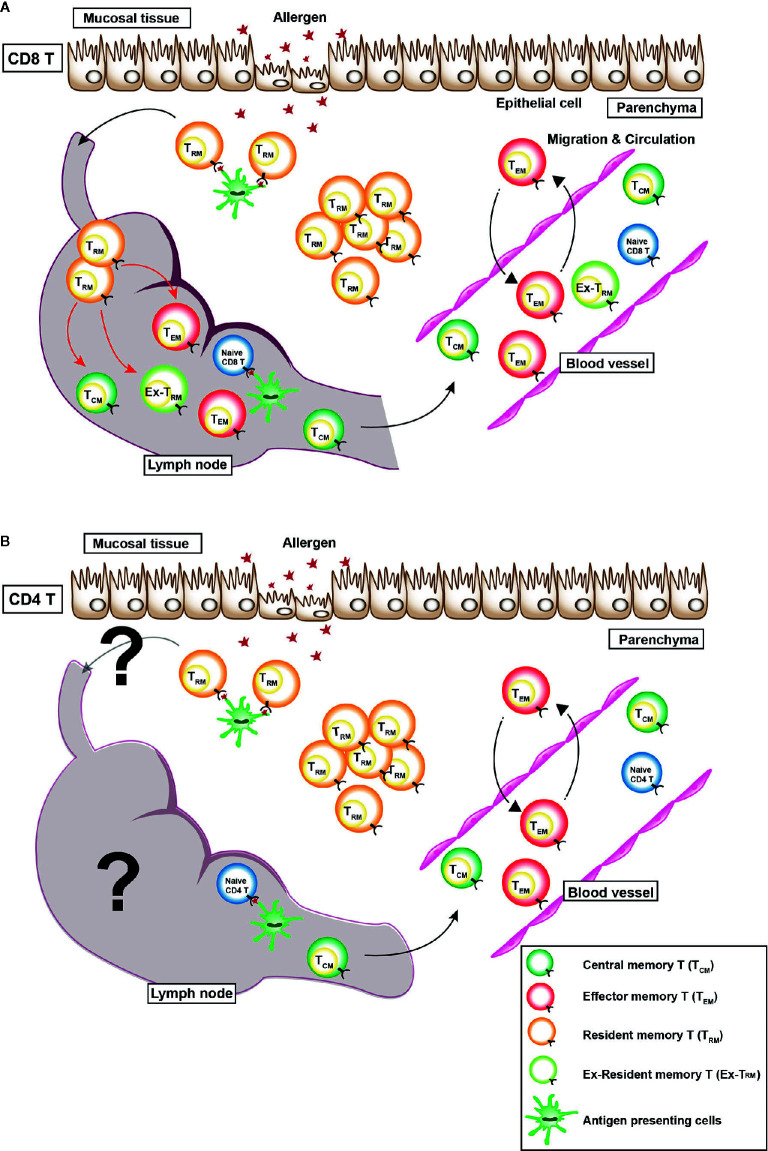
Distribution of various memory T cells *in vivo*. There are three types of memory T cells *in vivo*: (1) central memory T (T_CM_) cells, which mainly reside in secondary lymphoid tissues, (2) effector memory T (T_EM_) cells, which circulate in the blood, non-lymphatic tissues, and secondary lymphoid tissues, and (3) resident memory T (T_RM_) cells, which reside within non-lymphoid tissues. **(A)** A recent study revealed that CX3CR1^hi^ CD8^+^ T_EM_ cells are largely excluded from peripheral tissues after viral infection (9). In case of CD8^+^ T_RM_ cells, a series of recent studies clearly showed that re-activated CD8^+^ T_RM_ cells rejoined the circulating pool and proliferated in draining lymph nodes (red arrows). Some T_EM_ cells move back and forth between the blood vessel and parenchyma. **(B)** However, whether or not CD4^+^ T_RM_ cells rejoin the circulating pool and a re-activated in the draining lymph nodes is unclear.

Recently, non-circulating memory T cells have been identified, which are now referred to as tissue resident memory T (T_RM_) cells (10). T_RM_ cells show the high expression of C-type lectin-like molecule CD69 and integrin E subunit molecule CD103. T_RM_ cells produce various kind of cytokines, including IL-2, IFN-γ, TNF-α, and IL-17 (11–16). Unlike T_CM_ cells and T_EM_ cells, which circulate throughout the body *via* blood vessels and lymphatic vessels, T_RM_ cells do not circulate throughout the body, but they reside in non-lymphoid tissues such as the lung, skin, and gut. However, a series of recent studies clearly show that re-activated CD8^+^ T_RM_ cells rejoin the circulating pool and proliferate in draining lymph nodes (**Figure 1**) (17, 18). Regarding CD4^+^ T_RM_ cells, CD4^+^ T_RM_ cells account for 30% of the lymph node-CD4^+^ T cell population, which is a larger proportion than that of CD8^+^ T cells (19). However, the plasticity of subpopulations of memory CD4^+^ T cell has remained unclear. Regardless, the functions of memory T cells are closely linked to their mobility in the body of the host.

In mucosal tissues, such as the skin and female reproductive tract, antigen-recognized CD8^+^ T_RM_ cells produce IFN-γ and TNF-α to recruit other immune cells and activate dendritic cells and NK cells (12–14). In non-mucosal tissues, such as the brain and liver, CD8^+^ T_RM_ cells reside in each organ and play crucial roles in the host defense against pathogens (20, 21). In the brain, IFN-γ and Perforin-producing CD8^+^ T_RM_ cells act as an autonomous cytotoxic barrier to viral infection (21). In the lymphocytic choriomeningitis virus (LCMV)-infected brain, almost all CD8^+^ T_RM_ cells express CD69, but these cells show heterogeneous expression patterns of CD103 (21). In the liver, CXCR3^+^CD8^+^ T_RM_ cells are essential for protection against liver-stage malaria (20). Human CD69^+^CD103^+^CD8^+^ T_RM_ cells in the liver produce large amounts of IL-2 compared to CD69^-^CD103^-^CD8^+^ T cells (15).

Regarding CD4^+^ T cells, recent studies have highlighted prominent populations of CD4^+^ T_RM_ cells in various mucosal tissues, such as the skin (22–25), female genital tract (19, 22, 26, 27), small intestine (19, 28–30) and lung (16, 19, 22, 30–33). In the skin, CD4^+^ T_RM_ cells protect hosts against invading pathogens, including *Leishmania major* (23, 24). *Candida albicans* infection also induces IL-17-producing CD4^+^ T_RM_ cells in the skin (34). In the female genital tract, CD4^+^ T_RM_ cells are crucial for antiviral defense against genital herpes simplex virus 2 (HSV-2) infection (26, 27). Helminth infection and *Listeria monocytogenes* infection cause the induction of functional CD4^+^ T_RM_ cells in the intestine (28, 29). In the upper tract, pneumococcus infection induces CD4^+^ T_RM_ cells that prevent pneumococcal colonization (33). Furthermore, lung CD4^+^ T_RM_ cells are essential for protection against bacterial infection (16). Thus, similar to CD8^+^ T_RM_ cells, CD4^+^ T_RM_ cells may facilitate a rapid immune response to protect the host against re-exposure to pathogens in various mucosal organs.

In human, CCR7^hi^ CD4^+^ T_RM_ cells are detected in the female genital tract (35). In infants, mucosal memory CD4^+^ and CD8^+^ T cells already show characteristics of tissue residency, such as the enhanced expression of CD69 and CD103, which suggests that local *in situ* priming to antigens causes the induction of T_RM_ cells (36). Investigations of human samples from the lung after lung transplantation have revealed that lung-infiltrating recipient CD4^+^ and CD8^+^ T cells gradually acquire T_RM_ phenotypes, such as the enhanced expression of CD69 and CD103, over several months *in vivo* (37). In non-mucosal sites, human brain CD4^+^ T cells show the high expression of CD69 but a low expression of CD103 (38). More detailed information about human T_RM_ cells has been reviewed in other articles (39, 40). The roles of CD4^+^ T_RM_ cells in the non-mucosal tissue have not been well elucidated.

In addition to the essential role of T_RM_ cells in the biological defense of mucosal and non-mucosal organs, T_RM_ cells and other tissue resident immune cells, including innate lymphoid cells (ILCs), play a critical role in tissue homeostasis (41).

## The Molecular Mechanisms Underlying the Induction and Maintenance of the Tissue Residency of T_RM_ Cells

The mobility of T cells among various organs throughout the body is tightly regulated by various cytokines, chemokines and cell surface molecules (42). Transforming growth factor β (TGF-β) is an essential cytokine for the development of CD8^+^ T_RM_ cells in the mucosal tissues (43). TGF-β induces the expression of CD103 on CD8^+^ T cells (44). In the skin, CD8^+^ T_RM_ cells require transactivated autocrine TGF-β for epidermal persistence (45). An important cytokine for the survival of CD8^+^ T_RM_ cells in the skin is IL-15 (46). In the skin, hair follicle-derived IL-15 and IL-7 are required for the maintenance of CD8^+^ T_RM_ cells (47). During influenza viral infection, IFN-γ produced by CD4^+^ T cells induces CD8^+^ T_RM_ cells, which are crucial for protection against pathogenic viruses (44).

For the long-term survival of CD4^+^ T_RM_ cells, IL-7 is needed in the skin (47). In the lung, IL-15 is required for the generation of CD4^+^ T_RM_ cells (48).

Regarding chemokines and cell surface molecules, CD62L and CCR7 must be expressed on T cells to enter the peripheral lymph nodes (1), while Sphingosin-1-Phosphate Receptor 1 (S1P1), which binds the ligand Sphingosin-1-Phosphate (S1P), allows T cells to leave the lymph nodes and enter the lymphatic vessels (49). In humans, both CD8^+^ and CD4^+^ T_RM_ cells upregulate the adhesion molecules ITGAE (CD103) and ITGA1 (CD49a) as well as inhibitory molecules, including PD-1 and the dual specificity phosphatase DUSP6 (30). Both CD8^+^ and CD4^+^ T_RM_ cells show the down-regulated expression of S1PR1 (30). CD69 is a type 2 glycoprotein with a C-type lectin-like domain that acts as a homodimer (50). CD69 binds to S1P1 to promote the internalization and degradation of S1P1 in the cytoplasm. As a result, CD69-expressing T cells remain within lymphoid tissues, such as the thymus and lymph nodes (49). CD8^+^ T_RM_ cells in the lungs of mice with influenza viral infection show the high expression of CD69, and a CD69-deficient environment was shown to be associated with a reduced number of CD8^+^ T_RM_ cells in the lung (51, 52). In the skin and kidneys, CD69-deficiency in CD8^+^ T cells also result in a markedly reduced number of CD8^+^ T_RM_ cells (53, 54). CD8^+^ T_RM_ cells show lower S1P1 expression levels (43). In addition, CD8^+^ T_RM_ cells reveal the low expression of Krupple-like factor 2 (KLF2), a transcription factor that regulates the expression of S1PR1 (55). These findings suggest that CD69 plays a crucial role in CD8^+^ T_RM_ cells, as more than a mere cell surface marker. Interestingly, though, CD8^+^ T_RM_ cells are able to be maintained in the lung independently of the CD69 expression (52). Furthermore, experiments using pet mice with differing microbial experiences revealed that the CD69 expression on CD8^+^ T cells was insufficient to interpret tissue residence (56). Indeed, the functional requirement for CD69 is evidently dependent on the tissue where CD8^+^ T_RM_ cells exist (54). Thus, although CD69 is not a perfect cell surface marker for tissue residency, more detailed studies regarding the functional roles of CD69 in T_RM_ cells, especially CD8^+^ T_RM_ cells, are needed to draw firm conclusions. In contrast, the role of CD69 in CD4^+^ T_RM_ cells remains unclear.

The unique transcriptional features of T_RM_ cells have been well established in CD8^+^ T_RM_ cells. The transcription factor homolog of Blimp1 in T cells (Hobit) is specifically expressed in CD8^+^ T_RM_ cells (57). Hobit and Blimp1 cooperatively downregulate the expression of S1pr1 and Ccr7, which are required for tissue egress (57). Hobit and Blimp1 also repress the transcription factors Tcf7 and Klf2, which regulate survival and trafficking of circulating memory T cells (57). The transcription factor Runx3 plays a crucial role in establishing CD8^+^ T_RM_ cells (57, 58). CD8^+^ T_RM_ cells in the liver show an enhanced expression of *Hobit* (20). Without appropriate CD4^+^ T cell help, lung CD8^+^ T_RM_ cells show an enhanced expression of T-bet that suppresses the formation of CD8^+^ T_RM_ cells by direct binding to the *Itgae* locus (44).

Regarding CD4^+^ T cells, Hobit and Blimp1 are reported to attenuate CD4^+^ T_RM_ cell-dependent colitis (59). Viral infection induced-CD4 T_RM_ cells show the enhanced expression of Hobit and Eomes (19). However, another group reports that T helper type 2 (Th2) CD4 T_RM_ cells do not preferentially express Hobit, Blimp1 or Runx3 in their RNA sequencing (RNA-Seq) data sets (60). In humans, the transcription factor c-MAF induces the tissue residency transcriptional program in Th17 cells (61). Although many of the phenotypic characteristics of CD4^+^ T_RM_ cells are shared with CD8^+^ T_RM_ cells, precise assessments regarding the transcriptional features of CD4^+^ T_RM_ cells are required to identify the nature of CD4^+^ T_RM_ cells (62).

Recent studies using human tissue resident memory T cells have revealed that both CD4^+^ and CD8^+^ T_RM_ cells are transcriptionally distinct from other memory T cell subsets (30, 63). A core gene signature including ITGA1, ITGAE, IL-2, CXCR6, and PD-1 shows differential regulation between T_RM_ cells and circulating T cells, suggesting the unique feature of human T_RM_ cells *in vivo* (30).

## The Experimental Techniques Used to Identify T_RM_ Cells *In Vivo*

**Figure 2:**
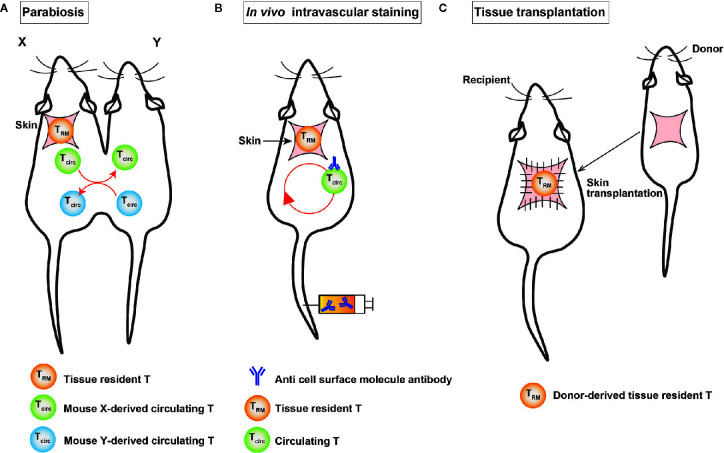
A schematic illustration of the experimental techniques used to identify T_RM_ cells. **(A)** Surgical connection of two congenic mice allows them to share blood circulation. **(B)** *In vivo* intravascular staining marks circulating T cells through the intravascular injection of an anti-cell surface molecule antibody. **(C)** In tissue transplantation, donor-derived T cells are detected in the graft after transplantation.

Proving the tissue residency of T cells is a major challenge. It is necessary to show at least that the cells are present in the same tissue for a certain period to prove tissue residency. Currently, experimental techniques, such as (1) parabiosis, (2) *in vivo* intravascular staining, and (3) tissue transplantation are used to prove the tissue residency of a certain population of cells (**Figure 2**).

Parabiosis is an experimental technique in which two mice are surgically linked and share a common circulatory system (**Figure 2**), which makes us possible to separate substances that are circulating in blood vessels and those that are not in the bloodstream. This method was established in France in the 19th century. In the second half of the 20th century, it has been widely used to investigate the endocrine system. In the field of immunology, parabiosis experiments are conducted to demonstrate the tissue residency of a certain cell population *in vivo*. In the tissue transplantation, the tissue—together with tissue-resident cells—is transplanted into congenic mice and then analyzed for the migration of donor-derived cells in the tissue to demonstrate tissue residency (10). Intravascular *in vivo* labeling is an experimental technique using the intravenous injection of cell-surface antibodies, such as anti-CD4 antibodies, to distinguish cells in tissue from those in blood vessels (**Figure 2**) (64). The advantage of this technique is its simplicity in comparison to parabiosis and tissue transplantation experiments. T cells in the vasculature were found to differ from those in the lung parenchyma, which were not stained with cell-surface antibodies (64). However, it is important to note that this experiment shows that unstained cells were not present in the vessels for a certain period of time after the intravenous injection of the antibody, because the cells were collected from each organ 3-5 minutes after the intravenous injection of the antibody under anesthesia.

As each of these techniques has certain limitations and addresses several specific criteria for residency, the definitive assessment of tissue residency of T cells should rely on supportive results obtained from multiple experimental techniques.

## The Protective and Pathogenic Roles of CD4^+^ T_RM_ Cells at Local Inflammatory Sites

In addition to other memory T cell populations, such as T_EM_ and T_CM_ cells, T_RM_ cells play an important role in the body’s defense against infection. In several experimental models in mice, CD8^+^ T_RM_ cells have been revealed to be important in defending against viral, parasitic and other infections (20, 65–67). In humans, CD8^+^ T_RM_ cells have been reported to be crucial in defending against herpes simplex type 1 virus infection in the skin (68).

Regarding CD4^+^ T cells, CD4^+^ T_RM_ cells are important for optimal protection against respiratory virus infection *via* the enhanced production of IFN-γ (11). CD4^+^ T_RM_ cells play key roles in the elimination of HSV-2 and chlamydia in the vagina (26, 69). HSV-2-specific CD4^+^ T_RM_ cells are enriched in local inflammatory sites, and the chemokine CCL5 is important for the retention of CD4^+^ T_RM_ cells in vaginal tissues (26). These CD4^+^ T_RM_ cells also produce large amounts of IFN-γ (26). In an LCMV infection model, CD4^+^ T_RM_ cells play a key role in local immunosurveillance along with CD8^+^ T_RM_ cells (19). CD4^+^ T_RM_ cells also play a protective role against pneumococcal infection in the lung (70). In this model, IL-17-producing CD4^+^ T_RM_ cells recruit neutrophils to the lung, which is crucial for protecting the host against bacterial infection (70). In humans, an increased frequency of donor T_RM_ cells in the lung of patients with lung transplantation is associated with a reduced rate of adverse clinical events, such as primary graft dysfunction (37). This finding suggests the protective roles of donor T_RM_ cells in the rejection of transplanted tissue.

However, T_RM_ cells are also involved in the pathogenesis of various human immune-related diseases. In psoriasis, an autoimmune disease of the skin, CD8^+^CD49a^-^ T_RM_ cells produce IL-17 at the local inflammatory site and are involved in the pathogenesis of the disease. In vitiligo, CD8^+^CD49a^+^ T_RM_ cells produce IFN-γ in the inflammatory tissue and are involved in the pathogenesis of the disease (71). In addition, using experimental autoimmune encephalomyelitis, a mouse model of multiple sclerosis, CD8^+^ T_RM_ cells have been shown to be involved in the onset and relapse of disease (72).

Mucosal tissues that include a large number of T_RM_ cells are susceptible to environmental stresses, such as cell damage, cell death, and changes in partial oxygen pressure. T_RM_ cells play important roles in maintaining local tissue homeostasis, including tissue repair and regeneration as well as defense against infection and the pathogenesis of immune-related diseases. Indeed, CD8^+^ T_RM_ cells localize within local inflammatory sites during tissue regeneration after influenza virus infection (52). This suggests that CD8^+^ T_RM_ cells are involved in the processes of tissue repair and regeneration. However, overactivation of the tissue repair process causes tissue fibrosis (73). Various stimuli, including HDM and fungal infection, cause fibrosis in the lung (73–75). In fact, house dust mite (HDM)-induced allergic airway inflammation has been demonstrated to be dependent on HDM antigen-specific CD4^+^ T_RM_ cells in the lungs in experimental mouse models (74, 76). IL-2 signaling is required for the residency of HDM antigen-specific CD4^+^ T_RM_ cells, which are sufficient to induce airway hyper-responsiveness (76). Interestingly, chronic exposure of HDM induces the infiltration of both CD4^+^ and CD8^+^ T cells into the lung tissue; however, only CD4^+^ T_RM_ cells persist in the lung for a long time (77). Another group reported that allergen-specific CD4^+^ T cells were able to survive for over 70 days in the lung (74). A dominant type 2 immune response is induced by repetitive HDM exposure, and Th2 T_RM_ cells are functionally and transcriptionally distinct from circulating memory Th2 cells in the lungs of mice with HDM-induced allergic inflammation (60). Th2 T_RM_ cells express increased levels of *Il5* and *Il13* (60). Thus, CD4^+^ T_RM_ cells play a critical role in shaping various pathologies, such as airway hyper-responsiveness and eosinophilic inflammation during chronic type 2 inflammation.

**Figure 3:**
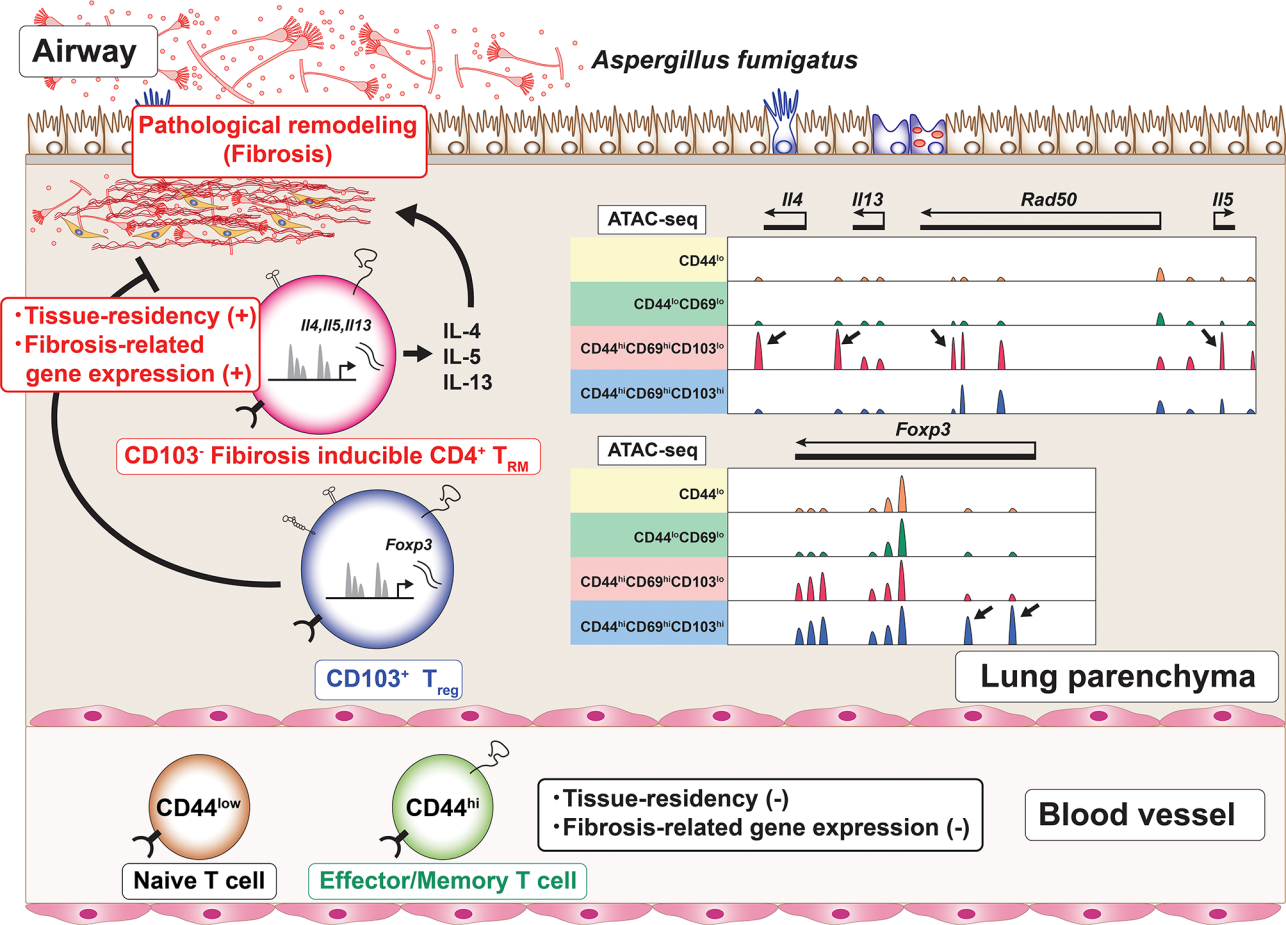
The induction of CD4^+^ T_RM_ cells with a unique regulome signature. Chronic allergic inflammation with fibrosis of the lung induced by repeated exposure to *Aspergillus fumigatus* antigen causes the induction of two cell populations, CD103-negative CD4^+^ tissue-resident memory T (T_RM_) cells and CD103-positive regulatory T (Treg) cells, which are involved in the pathogenesis of fibrotic responses. Each of these cell populations has its own characteristic regulome. For example, CD103-negative CD4^+^ T_RM_ cells produce proinflammatory cytokines and show specific peaks of ATAC-Seq in the Th2 cytokine loci (arrows). In contrast, CD103-positive Treg cells show specific peaks of ATC-Seq in the *Foxp3* locus (arrows).

Furthermore, Th2 T_RM_ cells show the enhanced expression of metalloproteases, extracellular matrix (ECM) components and regulators for ECM (60). These unique transcriptomic feature of Th2 T_RM_ cells suggests the pathogenic role of Th2 T_RM_ cells in the induction of fibrotic responses. Regarding fungal infection, patients with allergic bronchopulmonary aspergillosis/mycosis (ABPA/ABPM) have recurrent bronchial asthma attacks accompanied by bronchial dilatation and fibrotic changes in the lung (75). In the lungs of mice with repeated exposure to the *Aspergillus fumigatus* antigen, CD4^+^ T_RM_ cells, which produce various type of inflammatory cytokines accompanied by the low expression of CD103 and the enhanced expression of fibrosis-related genes, induce fibrotic responses (78). In addition, CD103^-^ CD4^+^ T_RM_ cells also express the metalloprotease *Adam8* (78). An assay for transposase-accessible chromatin using a sequencing (ATAC-Seq) analysis revealed that the characteristic features of these CD4^+^ T_RM_ cells populations were regulated at the chromatin level. For example, the regulatory elements of inflammatory cytokines, such as *Il4*, *Il5*, and *Il13*, were specifically accessible in CD103-negative CD4^+^ T_RM_ cells (**Figure 3**). At the same time, CD103-positive CD4^+^ regulatory T (Treg) cells are induced in the inflammatory lung. These CD103-positive Treg cells regulate the fibrotic responses induced by CD103-negative CD4^+^ T_RM_ cells in chronic allergic inflammation caused by repeated exposure to the *A. fumigatus* antigen *in vivo* (78) (**Figure 3**). Thus, CD103^-^ CD4^+^ T_RM_ cells are involved in the fibrotic response processes in the lung. Taken together, these findings suggest that CD4^+^ T_RM_ cells play pathogenic roles in the fibrosis induced by various stimuli, such as HDM and fungi.

The protective roles of CD4^+^ T_RM_ cells have been elucidated in various infectious diseases. However, the pathogenic roles of CD4^+^ T_RM_ cells in chronic inflammation other than type 2-related diseases, such as allergic inflammation, have been unclear. Thus, we await the further investigation of the pathogenic roles of CD4^+^ T_RM_ cells in various immune-related diseases, including multiple sclerosis and psoriasis, the induction of which reportedly involves type 17 inflammation.

## Plasticity and Epigenetics of T_RM_ Cells

It is now clear that memory T cells comprise several subsets, including T_CM_ cells, T_EM_ cells and T_RM_ cells. Researchers have shown that CD8^+^ T_CM_ cells become CD8^+^ T_RM_ cells *via* an adoptive transfer experimental system (79). In fact, adoptively transferred CD8^+^ T_CM_ cells reside in the skin of donor mice accompanied by the enhanced expression of CD69 and CD103 after viral infection (79).

But what about the opposite direction of re-differentiation? In other words, do CD8^+^ T_RM_ cells have the ability to re-differentiate to CD8^+^ T_CM_ cells? T_RM_ cells are localized within specific organs for a long time, indicating their involvement in first-line protective responses against local reinfection. If CD8^+^ T_RM_ cells can re-differentiate to CD8^+^ T_CM_ cells, T_RM_ cells may be involved in systemic memory immune responses. Experiments using CD8^+^ T_RM_ cells accompanied by an analysis of the methylation state of the CpG region have shown that the function of T_RM_ cells is not fixed, and T_RM_ cells have the ability to change their function *in vivo* (17). A machine learning-based analysis using the methylation state of the CpG region in CD8^+^ T_RM_ cells showed that CD8^+^ T_RM_ cells were able to re-differentiate (17). Furthermore, using an experimental system of virus-infected mice, researchers showed that some reactivated CD8^+^ T_RM_ cells returned to the systemic circulatory system and re-differentiated into CD8^+^ T_CM_ cells. Using a CD8^+^ T_RM_ cell-restricted transcription factor Hobit-reporter system, another group showed that Hobit^+^ CD8^+^ T_RM_ cells proliferate in draining lymph nodes after viral re-infection (18). Importantly, Hobit^+^ CD8^+^ T_RM_ cells re-differentiated into CD8^+^ T_EM_ cells together with the downregulation of the *Hobit* expression and contributed to the generation of the systemic immune responses (18). These results suggest that immune memory maintained in the local inflammatory sites may also be involved in systemic memory immune responses, at least in the case of CD8^+^ T_RM_ cells.

An IL-17A tracking-fate mouse experimental system showed that CD4^+^ T_RM_ cells were derived from effector Th17 cells (16). In humans, CD4^+^ T_RM_ cells in the bone marrow show unique DNA methylation profiles among memory T cell subsets, indicating their specialized function (80). However, in contrast to findings concerning CD8^+^ T cells, the plasticity of the CD4^+^ memory T cell population has remained unclear.

## The Maintenance of T_RM_ Cells in the Non-Lymphoid Tissue

Inducible bronchus-associated lymphoid tissue (iBALT), a type of ectopic lymphoid tissue, is often formed in response to various stimuli, including infection, smoking, and collagen disease, in the inflamed lung (81). iBALT includes MHC class II-positive cells, B220-positive cells, CD11c-positive cells, VCAM1-positive stromal cells, and CD21-positive follicular dendritic cells. CD11c-positive dendritic cells are crucial for the reactivation of CD8^+^ T_RM_ cells in the lung (82). Memory CD4^+^ T cells are maintained within iBALT in lungs with chronic allergic inflammation (83). Furthermore, Thy1-positive IL-7-producing lymphoid endothelial cells are essential for the survival of memory CD4^+^ T cells due to their production of IL-7 in the inflammatory tissue of the lung (83). Interestingly, the maintenance of allergen-specific CD4^+^ T cells is dependent on IL-7 signaling in the lung (74). Single-cell RNA sequencing of the lung from mice with bacterial infection has revealed the enhanced expression of *Il7* by lymphatic endothelial cells, which are colocalized with CD4^+^ T cells (16). Based on these findings, it is likely that CD4^+^ T_RM_ cells, which are induced by repeated exposure to *Aspergillus fumigatus* antigen, are also maintained within iBALT in the inflamed lung. In fact, repeated exposure to *Aspergillus fumigatus* antigen induces the enhanced formation of iBALTs in the inflamed lung. However, the molecular mechanisms underlying the differentiation, induction, and maintenance of CD4^+^ T_RM_ cells in the lung and the role of iBALT in these processes remain unclear and require further research. In another mucosal tissue, the skin, the formation of ectopic lymphoid tissue called inducible skin-associated lymphoid tissue (iSALT) was reported (84). CD4^+^ T_RM_ cells accumulate within iSALT following skin inflammation (84, 85). IL-7 is a key cytokine supporting the long-term survival of CD4^+^ T_RM_ cells in the skin (47).

More detailed information regarding the tissue-specific anatomical niches for the maintenance of CD4^+^ T_RM_ cells has been reviewed in other articles (62, 86).

## T_RM_ Cells and the “Pathogenic Th Cell Disease Induction Model”

We proposed a model for the pathogenesis of immune-related inflammatory diseases called the “pathogenic Th-cell disease induction model” (87). In our proposed “pathogenic Th-cell disease model”, a certain population of memory CD4^+^ T cells is highly pathogenic, and the generation of pathogenic T cells is important for the pathogenesis and regulation of various inflammatory diseases. In other words, various immune-related chronic inflammatory diseases are not induced by an imbalance between the subsets of CD4^+^ T cells (e.g., Th1 cells, Th2 cells or Th17 cells), rather, they are induced by a specific population of pathogenic cells (pathogenic CD4^+^ T cells) that arise in peripheral tissues under certain conditions. For example, we identified IL-5 high-producing-pathogenic Th2 cells that produce large amount of IL-5 and induce eosinophilic airway inflammation (88). We also identified fibrosis-inducing-pathogenic Th2 cells that produce Amphiregulin, a tissue repair factor, and induce tissue fibrosis *via* the activation of eosinophils (89, 90). These pathogenic Th2 cells have also been found in tissue, as they are maintained within the iBALT.

The CD103-negative CD4^+^ T_RM_ cells that we identified recently are also pathogenic CD4^+^ T cells, which coexist with pathogenic Th1/Th2/Th17 cells due to the nature of the pathological model of *Aspergillus fumigatus* antigen administration. Interestingly, both pathogenic CD4^+^ T_RM_ cells and regulatory T cells are induced simultaneously in chronic inflammatory tissues. Thus, multiple functional CD4^+^ T_RM_ cell populations are involved in the pathogenesis of refractory immune-related inflammatory diseases, such as bronchial asthma and atopic dermatitis. We need to investigate the diversity of CD4^+^ T_RM_ cells in the lung using a single cell RNA-sequencing (scRNA-seq) analysis.

## Closing Remarks

Tissue-resident memory T cells represent a relatively new cell population that has only been attracting attention for approximately 10 years. Regarding CD8^+^ T cells, the tissue-resident memory T cell population is being actively studied worldwide, and novel findings about CD8^+^ T_RM_ cells have emerged one after another, including the identification of transcription factors such as *Hobit*, *Blimp1*, and *Runx3*, which are important for the induction of CD8^+^ T_RM_ cells (57, 58). As described previously, the plasticity of CD8^+^ T_RM_ cells has also been analyzed at the epigenomic level.

On the other hand, the mechanisms underlying the differentiation, maintenance, and plasticity of CD4^+^ T_RM_ cells remain unclear. CD4^+^ T_RM_ cells play a protective role in the lungs against infections such as *Streptococcus pneumoniae* and *Mycobacterium tuberculosis* (70, 91). CD4^+^ T_RM_ cells also play an important role in the elimination of HSV-2 and chlamydia in the vagina (26). The intranasal administration of pneumococci induces IL-17-producing CD4^+^ T_RM_ cells that protect the host against pneumococcal colonization (33). Intranasal vaccination of influenza virus induced the accumulation of both CD4^+^ and CD8^+^ T_RM_ cells in the lung of mice (92). Moreover, intranasal vaccination with Venezuelan equine encephalitis replicons (VRP) encoding a severe acute respiratory syndrome coronavirus (SARS-CoV) CD4^+^ T cell epitope resulted in airway memory CD4^+^ T cell-dependent protection against SARS-CoV (93). In humans, increased frequencies of CD4^+^ T_RM_ cells in the airway are associated with surviving severe disease of SARS-CoV-2 infection (94). Furthermore, CD4^+^ T_RM_ cells may promote the generation of antibodies by B cells against pathogenic microorganisms in mucosal tissues, including the lung. In fact, a subpopulation of CD4^+^ T_RM_ cells promotes humoral responses in the lung after viral infection (95, 96). This subpopulation shows the follicular helper T (Tfh)-like phenotype, including a high expression of PD-1 and CXCR5 (95). The differentiation of this subpopulation depends on B cells and the intrinsic expression of Bcl6 (95). Importantly, Bcl6^hi^ CD4^+^ T_RM_ cells, which are colocalized with B cells in iBALT, promote local antibody production and help CD8^+^ T_RM_ cells *via* the enhanced production of IL-21 (95, 96). Thus, CD4^+^ T_RM_ cells are a promising target cell population in terms of the development of next-generation vaccine therapies (97). In the future, more intensive research on CD4^+^ T_RM_ cells is expected to reveal new cellular mechanisms and molecular mechanisms for CD4^+^ T_RM_ cells.

## Author Contributions

Writing, reviewing, and editing: KH, KK, AA, MK, and TN. All authors contributed to the article and approved the submitted version.

## Funding

This work was supported by the following grants: Ministry of Education, Culture, Sports, Science and Technology (MEXT Japan) Grants-in-Aid for Scientific Research (S) JP19H05650, (B) 20H03685, (C) 17K08876, 18K07164 and 19K16683; Practical Research Project for Allergic Diseases and Immunology (Research on Allergic Diseases and Immunology) from the Japan Agency for Medical Research and Development, AMED (Nos. JP20ek0410082, JP20ek0410060 and JP19ek0410045); AMED-PRIME, AMED (No. JP20gm6110005); AMED-CREST, AMED (No. JP20gm1210003); Mochida Memorial Foundation for Medical and Pharmaceutical Research, MSD Life Science Foundation, The Naito Foundation and Takeda Science Foundation.

## Conflict of Interest

The authors declare that the research was conducted in the absence of any commercial or financial relationships that could be construed as a potential conflict of interest.
